# Anti-NMDA Receptor Encephalitis and Vaccination

**DOI:** 10.3390/ijms18010193

**Published:** 2017-01-18

**Authors:** Hsiuying Wang

**Affiliations:** Institute of Statistics, National Chiao Tung University, Hsinchu 30010, Taiwan; wang@stat.nctu.edu.tw or hsiuyingwang@nctu.edu.tw; Tel.: +886-3-571-2121 (ext. 56813); Fax: +886-3-572-8745

**Keywords:** anti-NMDA receptor encephalitis, Japanese encephalitis, microRNA, vaccination

## Abstract

Anti-*N*-methyl-d-aspartate (Anti-NMDA) receptor encephalitis is an acute autoimmune neurological disorder. The cause of this disease is often unknown, and previous studies revealed that it might be caused by a virus, vaccine or tumor. It occurs more often in females than in males. Several cases were reported to be related to vaccination such as the H1N1 vaccine and tetanus/diphtheria/pertussis and polio vaccines. In this study, we reported an anti-NMDA receptor encephalitis case that may be caused by Japanese encephalitis vaccination. To investigate the association between anti-NMDA receptor encephalitis and vaccination, we analyzed the phylogenetic relationship of the microRNAs, which significantly regulate these vaccine viruses or bacteria, and the phylogenetic relationship of these viruses and bacteria. This reveals that anti-NMDA receptor encephalitis may be caused by Japanese encephalitis vaccination, as well as H1N1 vaccination or tetanus/diphtheria/pertussis and polio vaccinations, from the phylogenetic viewpoint.

## 1. Introduction

Anti-*N*-methyl-d-aspartate (Anti-NMDA) receptor encephalitis is an acute disorder that presents a multistage illness progressing from initial psychiatric symptoms to memory disturbances, seizures, dyskinesia and catatonia. In the cerebrospinal fluid (CSF) or serum of patients one can find antibodies produced by the body’s own immune system attacking *N*-methyl-d-aspartate (NMDA) receptors. Although this disorder may be induced by a virus, vaccination or tumor, the cause is often unknown. The treatments include first-line immunotherapies: steroids, intravenous immunoglobulin (IVIG) or plasmapheresis (or plasma exchange); and second-line immunotherapy such as rituximab or cyclophosphamide. It occurs more often in females than in males. A proportion of female patients have also been detected with ovarian tumors.

The recovery of anti-NMDA receptor encephalitis patients depends on the timeliness of the initiation of treatment, the choice of treatments and other factors. It has been reported that 79% of patients with anti-NMDA receptor encephalitis achieve a good outcome within 24 months of disease onset [1]. Case reports in the literature show a portion of patients fully recover within approximately one year. Studies on the efficacies of treatments for anti-NMDA receptor encephalitis have revealed that patients receiving at least two different forms of therapy may have higher efficacy rates than patients receiving a single form of therapy [2,3].

Since some patients have detectable tumors, especially ovarian tumors, and they had significant improvement after tumor resection, the tumor is a cause to this encephalitis. For those patients without detectable tumors, the cause is often unknown. Several anti-NMDA receptor encephalitis cases have been reported to be related to vaccination. Although vaccination is the most effective method of preventing infectious diseases, previous studies claim that vaccines cause chronic diseases such as asthma, multiple sclerosis, chronic arthritis, and diabetes [4]. Vaccination with live-attenuated polio vaccine occasionally results in the emergence of vaccine-derived polioviruses that may cause poliomyelitis [5]. The immunization induced by vaccination might cause autoimmune diseases because some microbial proteins are similar to human proteins [6]. As a result, the immune system might respond to self-proteins and cause damage to these proteins [7,8].

There have been several studies reporting that vaccination or virus may cause this disease. A 15-year-old female patient was diagnosed with anti-NMDA receptor encephalitis after a booster vaccination against tetanus, diphtheria, pertussis, and poliomyelitis [9]. Three patients developed the disorder after vaccination against H1N1 influenza, or after vaccination against tetanus, diphtheria, pertussis, and poliomyelitis [10]. In addition, herpes simplex encephalitis (HSE) was reported to be associated with anti-NMDA receptor encephalitis. A proportion of patients with HSE were shown to produce antibodies against NMDA receptors [11]. Two patients, an infant and an adult, had confirmed HSE and then developed confirmed anti-NMDA receptor encephalitis [12]. In this study, we report a case that had confirmed anti-NMDA receptor encephalitis after receiving a Japanese encephalitis (JE) vaccination. Three patients showed some evidence of double infection of JE virus and herpes simplex virus, and an experiment of double infection of JE virus and herpes simplex virus in mouse brain has been reported [13].

We analyzed nucleotide sequences of viruses, bacteria, and microRNAs (miRNAs) related to the vaccines in this study. miRNAs are small RNA molecules approximately 22 nucleotides long that can upregulate or downregulate their target gene expression post-transcriptionally [14]. The expression of miRNAs was shown to be altered in patients. As a result, miRNAs show potential to be prognostic or treatment response biomarkers of diseases. Microarray data analysis is a useful method to find miRNA biomarkers for diseases by comparing the expression profile between normal tissues and infected tissues or tumor tissues [15,16,17]. In addition, the phylogenetic analysis combined with the microarray data analysis can improve the accuracy of discovering miRNA biomarkers of diseases compared to using microarray data analysis alone [18].

To investigate the association between JE vaccination and anti-NMDA receptor encephalitis, we adopt a phylogenetic analysis to find the relationship of these viruses, bacteria, or related miRNAs of the vaccinations. The phylogenetic relationship of H1N1 virus, tetanus bacterium, diphtheria bacterium, poliomyelitis virus, herpes simplex virus, and JE virus are explored. In addition to the phylogenetic analysis of the virus (or bacteria) sequences, we also analyze the phylogenetic relationship of miRNAs that are related to H1N1, pertussis, poliomyelitis, HSE, JE, and anti-NMDA receptor encephalitis, respectively. Since we did not find studies of miRNAs related to tetanus or diphtheria, tetanus and diphtheria are not included in this miRNA analysis. Based on these phylogenetic analyses, the result reveals that anti-NMDA receptor encephalitis may be caused by JE vaccination, as well as H1N1 vaccination, or tetanus/diphtheria/pertussis and polio vaccination from the phylogenetic viewpoint.

## 2. Results

The literature revealed that H1N1 influenza vaccine, tetanus, diphtheria, pertussis, and poliomyelitis vaccine and HSE may associate with anti-NMDA receptor encephalitis. There has not been any report that has discussed the relationship between JE and anti-NMDA receptor encephalitis. In this study, we report an anti-NMDA receptor encephalitis case that is related to JE vaccination. Informed consent was obtained from the parents of this patient.

### 2.1. Case Report

We report about a two-year-old girl who was diagnosed with anti-NMDA receptor encephalitis. She developed a low-grade fever (38.2 °C) on Day 14 after receiving a second dose of JE vaccination. After taking medicine, she was fine until Day 17. She had a fever again (38.8 °C), and cried and vomited for three days. Psychiatric symptoms, including delusional features and talking nonsense, became apparent on Day 27. A blood test, abdominal ultrasonography, X-ray examination, and magnetic resonance imaging (MRI) showed no abnormalities, but the urinary tract was infected. IVIG was administrated. On Day 30, she was drooling in opisthotonus posturing and had an upward gaze and neck stiffness. Then she was transferred to an intensive care unit (ICU), and succumbed to unconsciousness and involuntary movements in the ICU. Anti-NMDA receptor encephalitis was confirmed by the detection of anti-NMDAR antibodies in CSF.

### 2.2. Analysis

To investigate the association between JE vaccination and anti-NMDA receptor encephalitis, the miRNAs related to H1N1, pertussis, poliomyelitis, HSE, JE, and anti-NMDA receptor encephalitis in human patients are presented in Table 1. The sequence accession numbers of these virus (or bacterium) are provided in Table 2. In this analysis, tetanus and diphtheria are not taken into account because we did not find miRNAs related to tetanus or diphtheria in the literature.

We give the details of Table 1. The involvement of miRNAs during influenza viral infection was unknown until 2010 in that miR-323, miR-491, and miR-654 were shown to inhibit replication of the H1N1 influenza A virus through binding to the *PB1* gene [19]. In addition, for the H1N1 influenza A vaccine, studies revealed that miR-let-7c was highly up-regulated in influenza virus infected A549 cells [20]; microarray analysis revealed that miR-31, miR-29a and miR-148a play an important role in patients infected with H1N1 influenza virus [21]; miR-146a was shown to be related to the outcome of influenza infection, and let-7f was downregulated in H1N1-infected cells [22].

For the pertussis vaccine, a panel of five miRNAs (miR-202, miR-342-5p, miR-206, miR-487b, and miR-576-5p) was confirmed to be overexpressed compared with the healthy control group [23]. For the poliomyelitis vaccine, miR-555 was found to have the most potent antiviral activity against three different oral polio-attenuated vaccine strains tested [5]. For herpes simplex virus (HSV), miR-145 regulated oncolytic HSV-1 is a promising agent for the treatment of non-small cell lung cancer [29]; HSV-1-induced miR-101 is mainly derived from its precursor hsa-mir-101-2, and the HSV-1 immediate early gene *ICP4* directly binds to the hsa-mir-101-2 promoter to activate its expression [24].

For Japanese encephalitis virus (JEV), miR-19b-3p and miR-155 were shown in regulating the JEV-induced inflammatory response [25,26]; miR-146a suppresses the cellular immune response in human microglial cells during JEV infection [27], and miR-33a-5p contributes to viral replication by targeting eukaryotic translation elongation factor 1A1 during JEV infection [30]. For the miRNAs associated with anti-NMDA receptor encephalitis, the let-7 family has been discovered in the literature, and the expression levels of let-7a, let-7b, let-7d, and let-7f were shown to be significantly downregulated in anti-NMDAR encephalitis compared with the negative controls [28].

The aim of this study is to explore the connection between these vaccines such that the cause of anti-NMDA receptor encephalitis related to these vaccines can be disclosed. Therefore, we analyze the relationship of these miRNAs and virus sequences or bacterium sequences. A basic and useful tool for exploring the relationship of nucleotide sequences is to study the phylogeny of these sequences, which is based upon similarities and differences in their physical or genetic characteristics. The phylogenetic trees of these sequences can be obtained based on different evolutionary models and distance methods. Although the phylogenetic analysis may not directly explain the relationship between pathogenetic mechanisms and miRNA (or mRNA), it can be used as a useful ancillary tool to explore their biological mechanisms.

**Figure 1:**
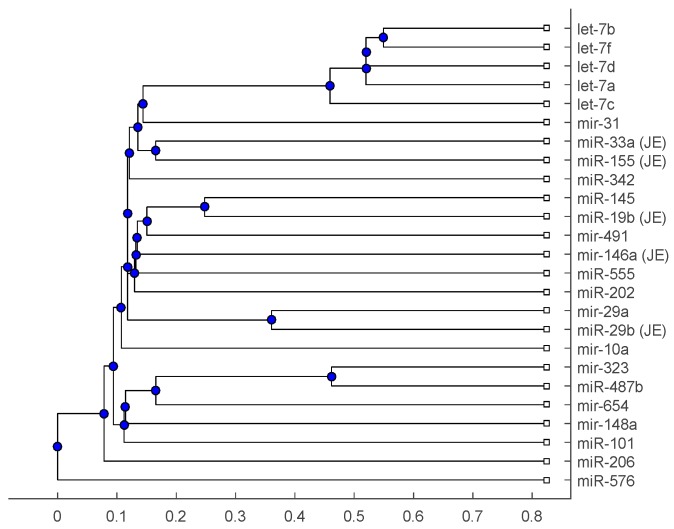
The phylogenetic tree of miRNAs based on the Juke–Cantor distance and single linkage function.

**Figure 2:**
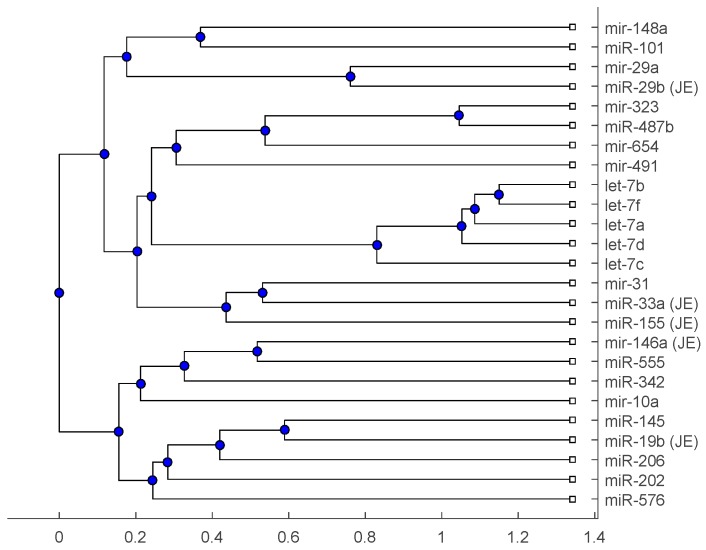
The phylogenetic tree of miRNAs based on the alignment score distance and average linkage function.

**Figure 3:**
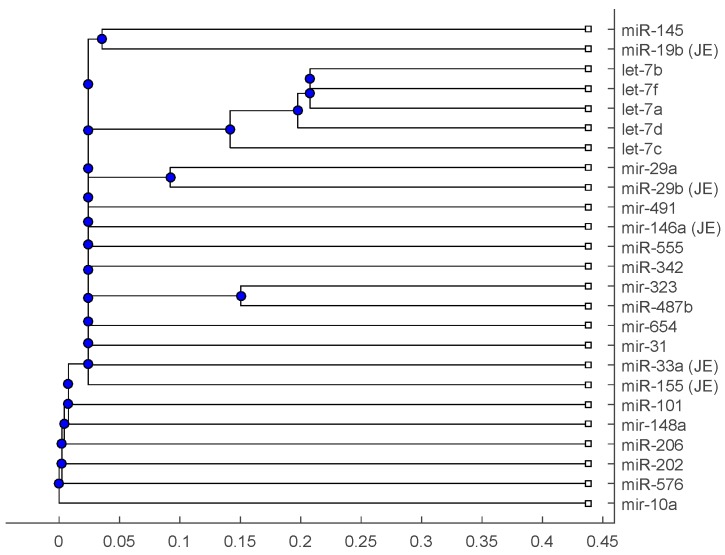
The phylogenetic tree of miRNAs based on the p-distance and median linkage function.

**Figure 4:**
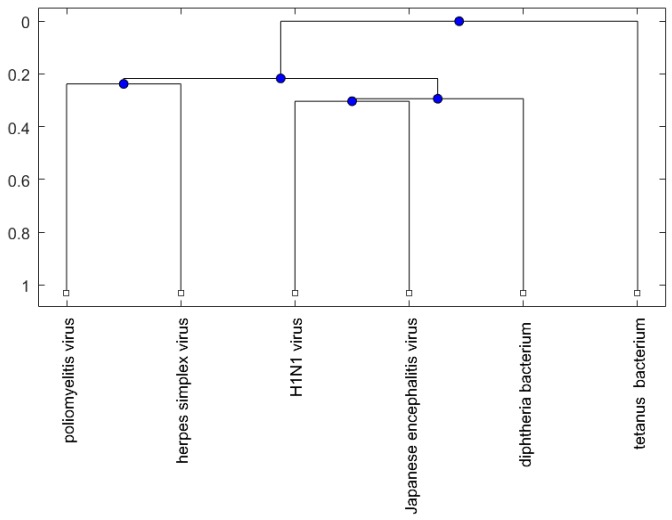
The phylogenetic tree of virus or bacterium based on the Juke–Cantor distance and single linkage function.

**Figure 5:**
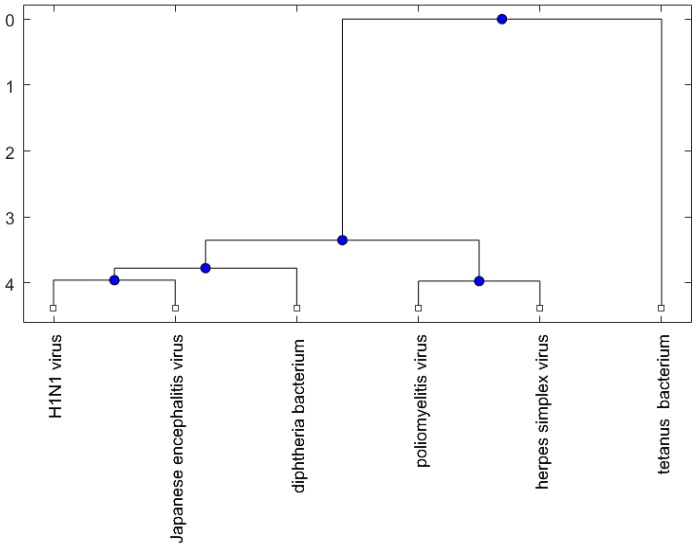
The phylogenetic tree of virus or bacterium based on the alignment score distance and average linkage function.

**Figure 6:**
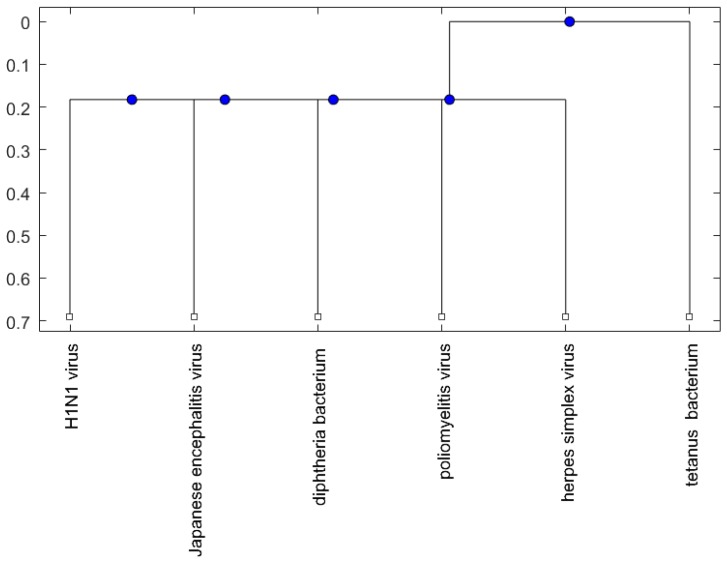
The phylogenetic tree of virus or bacterium based on the p-distance and median linkage function.

Figure 1, Figure 2 and Figure 3 show phylogenetic trees of the miRNAs for H1N1, pertussis, poliomyelitis, HSE, JE, and anti-NMDA receptor encephalitis based on different evolutionary models and distance methods; Figure 4, Figure 5 and Figure 6 show phylogenetic trees of sequences for H1N1, tetanus, diphtheria, poliomyelitis, herpes simplex virus, and JE virus using different evolutionary models and distance methods.

## 3. Discussion

The let-7 family was reported to be related to anti-NMDA receptor encephalitis [2,28]. let-7f is the only common miRNA of vaccine and anti-NMDA receptor encephalitis from Table 1. We might conclude that H1N1 vaccination has the largest chance of inducing anti-NMDA receptor encephalitis among these vaccinations. From Table 1, there is a common miRNA, miR-146, of H1N1 and JE. In addition, miR-29a for H1N1 and miR-29b for JE are in the miR-29 family. This may indicate JE vaccine is more relevant to H1N1 vaccine than to other vaccines.

The phylogenetic tree in Figure 1 shows miR-33a is nearest to the let-7 family among the five JE-related miRNAs, followed by miR-155. The situation is similar in Figure 2. Although miR-29b, miR-19b, and miR-146a are not very close to the let-7 family, compared with several miRNAs associated with other viruses (or bacteria), we may conclude that these two miRNAs are not very distinct from the let-7 family. For the phylogenetic tree in Figure 3, the five JE-related miRNAs belong to a large clade with the let-7 family.

In summary, for the miRNA comparison, from Figure 1, Figure 2 and Figure 3, the five miRNAs associated with the JE vaccine are closer to the let-7 family compared with the miRNAs associated with some other viruses (or bacteria). Although this phylogenetic analysis cannot directly infer that JEV vaccination might induce anti-NMDA receptor encephalitis, it shows a chance that the JE virus might cause the anti-NMDA receptor encephalitis by comparing with the other viruses (or bacteria) from the phylogenetic viewpoint.

For the viruses (or bacteria) comparison, from Figure 4, Figure 5 and Figure 6, the JE virus is always grouped together with H1N1 virus. Since two patients developing this disorder after vaccination against H1N1 influenza have been reported [10], there is a chance that the anti-NMDA receptor encephalitis is caused by the JE vaccination. In addition, JE vaccines have been reported to be linked to some fatal or serious events from the literature. A sudden death of a 10-year-old boy after JE vaccination was reported [31]. Several patients developed serious neurological symptoms within a few weeks after JE vaccination [32]. Two children developed acute disseminated encephalomyelitis (ADEM) after Japanese B encephalitis vaccination [33]. A five-year-old Japanese boy also developed ADEM two weeks after JE vaccination [34].

## 4. Methods and Materials

The molecular relationship for the viruses, bacteria, and miRNAs, which are related to anti-NMDA receptor encephalitis, HSE, the JE vaccine, tetanus/diphtheria/pertussis, polio, and the H1N1 influenza vaccine, are presented in terms of the phylogenetic tree method. There are a number of well-known methods to construct phylogenetic trees [35]. We plot the trees to analyze the relationships of these nucleotide sequences. Using these relationships, the chance of anti-NMDA receptor encephalitis caused by JE can be investigated.

The phylogenetic analysis of miRNAs is useful in discovering miRNA biomarkers for diseases [18]. To plot the phylogenetic tree of miRNAs in Table 1, we accessed the miRNA stem-loop sequences from miRBase [36]. For example, the accession number of Homo sapiens miR-155 (hsa-mir-155) is MI0000681, and its stem-loop sequence is CUGUUAAUGCUAAUCGUGAUAGGGGUUUUUGCCUCCAACUGACUCCUACAUAUUAGCAUUAACAG.

The stem-loop sequence of a precursor miRNA, including the 5p mature miRNA sequence and 3p mature miRNA sequence, can provide more information of miRNAs than only using mature miRNA sequences.

The phylogenetic trees are plotted using the Bioinformatics Toolbox of MATLAB [37]. To plot phylogenetic trees, we need to select a distance method to calculate the pairwise distances between two sequences and to select a linkage method to build a tree. Since the topologies of phylogenetic trees depend on the distance method and the linkage method, we plot several phylogenetic trees for the miRNAs and viruses (or bacteria), based on different distance methods and the linkage methods, respectively. Two MATLAB codes, *seqpdist* and *seqlinkage*, are used to plot the trees. For the miRNA phylogenetic trees, Figure 1 is the tree based on the Juke–Cantor distance and single linkage function; Figure 2 is the tree based on alignment score distance and average linkage function; and Figure 3 is the tree based on p-distance and median linkage function. By similar approaches, we can obtain three phylogenetic trees of these viruses (or bacteria) (Figure 4).

The sequences in the same clade are considered to have a similar phylogenetic structure. Although the structures of these trees may not be the only approach to analyze molecular relationships of these sequences, they can provide an ancillary tool to clarify whether vaccination is a likely cause of this disease.

## 5. Conclusions

Although there is few anti-NMDA receptor encephalitis cases reported to be related to vaccination, there may be more cases caused by vaccination that have not been reported. This study provides a JE vaccination-related case. Since there has not been any JE vaccination-related case reported in the literature, it is worth investigating the association between JE vaccination and anti-NMDA receptor encephalitis. Although the cause of anti-NMDA receptor encephalitis is still not very clear in this stage, more disclosures of the vaccination-related case can provide useful information for anti-NMDA receptor encephalitis management and prevention.

## Figures and Tables

**Table 1:** miRNAs related to vaccination or encephalitis.

Vaccine or Encephalitis	MircoRNA	References
H1N1	miR-323, miR-491, miR-654, miR-10a, let-7c, let-7f, miR-31, miR-29a, miR-148a, miR-146a	[19,20,21,22]
pertussis	miR-202, miR-342, miR-206, miR-487b, miR-576	[23]
poliomyelitis	miR-555	[5]
herpes simplex virus	miR-145, miR-101	[21,24]
Japanese encephalitis virus	miR-19b-3p, miR-33a-5p, miR-155, miR-29b, miR-146a	[25,26,27]
Anti-NMDA receptor encephalitis	let-7a, let-7b, let-7d, and let-7f	[28]

**Table 2:** Sequences of virus (or bacterium).

Vaccine	Virus (or Bacterium) Sequence Accession Number
H1N1	AF250365
Tetanus	X04436
Diphtheria	K01722
Poliomyelitis	KC880521
Herpes simplex virus	M38699.1
Japanese encephalitis virus	FJ938222

## References

[1] Titulaer M.J., McCracken L., Gabilondo I., Armangué T., Glaser C., Iizuka T., Honig L.S., Benseler S.M., Kawachi I., Martinez-Hernandez E. (2013). Treatment and prognostic factors for long-term outcome in patients with anti-NMDA receptor encephalitis: An observational cohort study. Lancet Neurol..

[2] Wang H. (2016). Efficacies of treatments for anti-NMDA receptor encephalitis. Front. Biosci..

[3] Duan B.C., Weng W.C., Lin K.L., Wong L.C., Li S.T., Hsu M.H., Lin J.J., Fan P.C., Lin M.I., Chiu N.C. (2016). Variations of movement disorders in anti-*N*-methyl-d-aspartate receptor encephalitis: A nationwide study in Taiwan. Medicine.

[4] Offit P.A., Hackett C.J. (2003). Addressing parents’ concerns: Do vaccines cause allergic or autoimmune diseases?. Pediatrics.

[5] Shim B.S., Wu W., Kyriakis C.S., Bakre A., Jorquera P.A., Perwitasari O., Tripp R.A. (2016). MicroRNA-555 has potent antiviral properties against poliovirus. J. Gen. Virol..

[6] Steinman L. (2001). Multiple sclerosis: A two-stage disease. Nat. Immunol..

[7] Regner M., Lambert P.H. (2001). Autoimmunity through infection or immunization?. Nat. Immunol..

[8] Albert L.J., Inman R.D. (1999). Mechanisms of disease: Molecular mimicry and autoimmunity. N. Engl. J. Med..

[9] Hofmann C., Baur M.O., Schroten H. (2011). Anti-NMDA receptor encephalitis after TdaP-IPV booster vaccination: Cause or coincidence?. J. Neurol..

[10] Dalmau J., Lancaster E., Martinez-Hernandez E., Rosenfeld M.R., Balice-Gordon R. (2011). Clinical experience and laboratory investigations in patients with anti-NMDAR encephalitis. Lancet Neurol..

[11] Mohammad S.S., Sinclair K., Pillai S., Merheb V., Aumann T.D., Gill D., Dale R.C., Brilot F. (2014). Herpes simplex encephalitis relapse with chorea is associated with autoantibodies to *N*-methyl-d-aspartate receptor or dopamine-2 receptor. Mov. Disord..

[12] DeSena A., Graves D., Warnack W., Greenberg B.M. (2014). Herpes simplex encephalitis as a potential cause of anti-*N*-methyl-d-aspartate receptor antibody encephalitis report of 2 cases. JAMA Neurol..

[13] Hayashi K., Arita T. (1977). Experimental double infection of Japanese encephalitis-virus and herpes-simplex virus in mouse-brain. Jpn. J. Exp. Med..

[14] Chuang J.C., Jones P.A. (2007). Epigenetics and microRNAs. Pediatr. Res..

[15] Wang H. (2014). Predicting cancer-related miRNAs using expression profiles in tumor tissue. Curr. Pharm. Biotechnol..

[16] Hsieh W.J., Lin F., Huang H., Wang H. (2014). Investigating microRNA-Target interaction-supported tissues in human cancer tissues based on miRNA and target gene expression profiling. PLoS ONE.

[17] Taguchi Y.H. (2016). Identification of more feasible microRNA–mRNA interactions within multiple cancers using principal component analysis based unsupervised feature extraction. Int. J. Mol. Sci..

[18] Wang H. (2016). Predicting microRNA biomarkers for cancer using phylogenetic tree and microarray analysis. Int. J. Mol. Sci..

[19] Song L., Liu H., Gao S., Jiang W., Huang W. (2010). Cellular microRNAs inhibit replication of the H1N1 influenza A virus in infected cells. J. Virol..

[20] Ma Y.J., Yang J., Fan X.L., Zhao H.B., Hu W., Li Z.P., Yu G.C., Ding X.R., Wang J.Z., Bo X.C. (2012). Cellular microRNA let-7c inhibits M1 protein expression of the H1N1 influenza A virus in infected human lung epithelial cells. J. Cell. Mol. Med..

[21] Song H., Wang Q., Guo Y., Liu S., Song R., Gao X., Dai L., Li B., Zhang D., Cheng J. (2013). Microarray analysis of microRNA expression in peripheral blood mononuclear cells of critically ill patients with influenza A (H1N1). BMC Infect. Dis..

[22] Terrier O., Textoris J., Carron C., Marcel V., Bourdon J.C., Rosa-Calatrava M. (2013). Host microRNA molecular signatures associated with human H1N1 and H3N2 influenza A viruses reveal an unanticipated antiviral activity for miR-146a. J. Gen. Virol..

[23] Ge Y.Y., Zhao K., Qi Y., Min X., Shi Z., Qi X., Shan Y., Cui L., Zhou M., Wang Y. (2013). Serum microRNA expression profile as a biomarker for the diagnosis of pertussis. Mol. Biol. Rep..

[24] Wang X., Diao C., Yang X., Yang Z., Liu M., Li X., Tang H. (2016). ICP4-induced miR-101 attenuates HSV-1 replication. Sci. Rep..

[25] Ashraf U., Zhu B., Ye J., Wan S., Nie Y., Chen Z., Cui M., Wang C., Duan X., Zhang H. (2016). MicroRNA-19b-3p modulates Japanese encephalitis virus-mediated inflammation via targeting RNF11. J. Virol..

[26] Thounaojam M.C., Kundu K., Kaushik D.K., Swaroop S., Mahadevan A., Shankar S.K., Basu A. (2014). MicroRNA 155 regulates Japanese encephalitis virus-induced inflammatory response by targeting Src homology 2-containing inositol phosphatase 1. J. Virol..

[27] Sharma N., Verma R., Kumawat K.L., Basu A., Singh S.K. (2015). miR-146a Suppresses cellular immune response during Japanese encephalitis virus JaOArS982 strain infection in human microglial cells. J. Neuroinflamm..

[28] Zhang J., Xu X., Zhao S., Gong Z., Liu P., Guan W., He X., Wang T., Peng T., Teng J. (2015). The expression and significance of the plasma let-7 family in anti-*N*-methyl-d-aspartate receptor encephalitis. J. Mol. Neurosci..

[29] Li J.M., Kao K.C., Li L.F., Yang T.M., Wu C.P., Horng Y.M., Jia W.W., Yang C.T. (2013). MicroRNA-145 regulates oncolytic herpes simplex virus-1 for selective killing of human non-small cell lung cancer cells. Virol. J..

[30] Chen Z., Ye J., Ashraf U., Li Y., Wei S., Wan S., Zohaib A., Song Y., Chen H., Cao S. (2016). MicroRNA-33a-5p modulates Japanese encephalitis virus replication by targeting eukaryotic translation elongation factor 1A1. J. Virol..

[31] Bunai Y., Ishii A., Akaza K., Nagai A., Nishida N., Yamaguchi S. (2015). A case of sudden death after Japanese encephalitis vaccination. Leg. Med. (Tokyo).

[32] Plesner A.M., Arlien-Soborg P., Herning M. (1998). Neurological complications to vaccination against Japanese encephalitis. Eur. J. Neurol..

[33] Ohtaki E., Murakami Y., Komori H., Yamashita Y., Matsuishi T. (1992). Acute disseminated encephalomyelitis after Japanese B encephalitis vaccination. Pediatr. Neurol..

[34] Ohya T., Nagamitsu S., Yamashita Y., Matsuishi T. (2007). Serial magnetic resonance imaging and single photon emission computed tomography study of acute disseminated encephalomyelitis patient after Japanese encephalitis vaccination. Kurume Med. J..

[35] Nei M., Kumar S., Ebrary Inc. (2000). Molecular Evolution and Phylogenetics.

[36] Kozomara A., Griffiths-Jones S. (2014). miRBase: Annotating high confidence microRNAs using deep sequencing data. Nucleic Acids Res..

[37] (2012). MATLAB and Bioinformatics Toolbox Release 2012b.

